# Opioid Utilisation in Hungary: National and Regional Analysis in Ambulatory and Hospital Care Sector

**DOI:** 10.3390/jcm14030897

**Published:** 2025-01-29

**Authors:** Ni Made Amelia Ratnata Dewi, Mária Matuz, Délia Szok, Zsófia Engi, Gyöngyvér Soós, Melinda Csenki, Emese Csüllög, Attila Balog, Dezső Csupor, Réka Viola, Ria Benkő

**Affiliations:** 1 Institute of Clinical Pharmacy, Faculty of Pharmacy, University of Szeged, 6725 Szeged, Hungary; matuz.maria@szte.hu (M.M.); engi.zsofia@szte.hu (Z.E.); soos.gyongyver@semmelweis.hu (G.S.); csupor.dezso@szte.hu (D.C.); tothne.viola.reka@szte.hu (R.V.); 2 Pharmacy Study Program, Faculty of Medicine and Health Sciences, University of Mataram, Mataram 83115, Indonesia; 3 Central Pharmacy Department, Albert Szent-Györgyi Health Center, University of Szeged, 6725 Szeged, Hungary; 4 Department of Neurology, Albert Szent-Györgyi Health Center, University of Szeged, 6725 Szeged, Hungary; szok.delia@med.u-szeged.hu; 5 Department of Oncotherapy, Albert Szent-Györgyi Health Center, University of Szeged, 6725 Szeged, Hungary; csenki.melindda@med.u-szeged.hu; 6 Department of Anesthesiology and Intensive Care, Albert Szent-Györgyi Health Center, University of Szeged, 6725 Szeged, Hungary; csullog.emese@med.u-szeged.hu; 7 Department of Rheumatology and Immunology, Albert Szent-Györgyi Health Center, University of Szeged, 6725 Szeged, Hungary; balog.attila@med.u-szeged.hu; 8 Institute for Translational Medicine, Medical School, University of Pécs, 7624 Pécs, Hungary; 9 Emergency Department, Albert Szent-Györgyi Health Center, University of Szeged, 6725 Szeged, Hungary

**Keywords:** ambulatory, hospital, Hungary, opioid, pharmacoepidemiology, utilisation

## Abstract

**Background/Objectives**: Opioid consumption analysis in Hungary, particularly through ambulatory and hospital sales data, including regional information, is lacking. This study examines opioid use in both sectors, explores regional variations, and identifies influencing factors. **Methods:** A cross-sectional analysis was conducted using sales data from ambulatory and hospital care, quantifying opioid consumption in defined daily doses (DDD) per 1000 inhabitants (DID) and per day, or DDD per 100 patient days (DHPD) at national and regional levels. Correlations between opioid utilisation and regional variables were assessed using Spearman’s rank test. **Results:** Total opioid use has risen from 4.73 DID in 2012 to 6.75 DID in 2021, with weak and oral opioids being the most used. Ambulatory care experienced significant increases in weak (61.48%) and oral opioid use (60.01%). Hospital care experienced a decline in DID and stagnation in DHPD. Tramadol combinations grew notably in ambulatory care, with tramadol-paracetamol rising from 0.37 DID to 2.17 DID (484.61% increase) and tramadol-dexketoprofen from 0.12 DID to 0.91 DID (650.27% increase). Interregional differences showed a maximum to minimum ratio of 1.79 in ambulatory and 3.03 in hospital care in 2021. Positive correlations were found between opioid use and geriatric population percentage (r = 0.475; *p* = 0.035) and, also, unemployment rate (r = 0.546; *p* = 0.014). A moderate negative correlation was observed between the number of general practitioners (r = −0.458; *p* = 0.043) and ambulatory care opioid use. **Conclusions:** Opioid use is increasing in Hungarian ambulatory care while remaining steady in the hospital sector. Regional variations are possibly linked to demographic and economic factors in ambulatory care.

## 1. Introduction

Pain greatly affects individuals, often resulting in anxiety, emotional turmoil, physical limitations, and a reduced quality of life [1]. Poor pain management may lead to higher treatment expenses and extended hospital admissions, creating an economic strain on healthcare systems and society [2]. Opioid analgesics offer a solution for alleviating both acute and chronic pain [3].

In certain countries, stringent opioid prescribing regulations have resulted in inadequate use of opioids for pain management [4,5]; however, in other countries, opioids are used excessively for treating acute and chronic pain [6,7,8]. The misuse of opioids has raised significant concerns, mainly because their long-term effectiveness is limited [9]. Long-term and excessive opioid use raises the risk of side effects like constipation, nausea, and dizziness. Additionally, overusing opioids increases the likelihood of requiring larger doses or more potent opioids, which could result in opioid use disorder [10]. In the USA, the opioid crisis over the past few decades has led to a notable rise in deaths from opioid overdoses [11].

A worldwide observational study revealed considerable variation in opioid usage rates [12]. Opioid usage rates were elevated in high-income countries, demonstrating a median consumption of 345.1 morphine milligram equivalents (MMEs) per 1000 inhabitants daily. In contrast, upper-middle-income countries had a median of 23.6 MMEs, while lower-middle-income countries showed an even lower median of 8.3 MMEs [12]. There has been a rising trend in opioid use in wealthier nations like Ireland [13], Denmark [14], Australia [15], Taiwan [16], Norway, Finland, and Iceland [17], and lower-income countries, such as Egypt [18], Malaysia, Indonesia, Thailand, and Vietnam [19]. Bosetti et al. carried out research utilising data from the International Narcotics Control Board (INCB) to examine opioid usage patterns across 22 European countries. Their findings revealed rising trends in opioid consumption [20]. Bäckryd et al. argue that the methodology employed in INCB reports results in an inflated estimate of opioid consumption, thus failing to accurately reflect the use of medical opioids [21]. Instead, it employs a non-standardised metric called defined daily doses for statistical purposes (S-DDD). This indicated that fentanyl constituted 68%–70% of the overall opioid consumption, whereas national sales data showed fentanyl use represented merely 3–6% of total opioid use, as reported in the World Health Organisation (WHO) defined measurement unit (defined daily dose—DDD) [21].

Engi et al. previously examined opioid usage in Hungary’s ambulatory care sector using reimbursement data. They discovered that, while opioid utilisation rose from 2006 to 2020, it was still regarded as underused [4]. However, Engi et al. may have slightly underestimated opioid usage because their study relied on reimbursement data. Moreover, there has been no analysis of opioid use in hospital care or at a regional level in Hungary. The purpose of this study is to estimate opioid use in both healthcare sectors, to reveal intra-country differences, and to identify potential determinants of regional opioid use.

## 2. Materials and Methods

### 2.1. Study Design

A cross-sectional study was conducted over a 10-year period, using retrospective data collection for the years between 2012 and 2021.

### 2.2. Settings and Data

This study used aggregated national and regional level data on the legal sale of medical opioids in both hospital and ambulatory care sector. In Hungary, ambulatory care services encompass drug use in nursing homes and prescriptions from private healthcare providers.

This study categorised opioids according to the WHO’s ATC index (2023 version), including all substances within N02A. We obtained data on 15 opioid analgesics from the N02A drug group available in Hungary, which includes fixed combination products: morphine, hydromorphone, oxycodone, combinations of oxycodone, pethidine, fentanyl, buprenorphine, nalbuphine, tapentadol, dihydrocodeine, codeine with paracetamol, tramadol with paracetamol, and tramadol with dexketoprofen.

The opioids were categorised based on their administration route and analgesic potency, namely strong and weak opioids. The weak opioids include dihydrocodeine, codeine, their combinations, tramadol, and tramadol combination products. Strong opioids consist of morphine, hydromorphone, oxycodone and its combinations, pethidine, fentanyl, buprenorphine, nalbuphine, and antispasmodics. Tapentadol is also considered a strong opioid [22]. The administration routes included oral, transdermal, rectal, and parenteral formulations. In the case of buprenorphine and fentanyl transdermal patches, the actual amount of opioid used was calculated by considering the hourly release rate of the active ingredient along with the intended use period of 72 h [23,24]. Remaining amounts of active agents in transdermal patches after the recommended and assumed 72-h usage were not included in the calculations.

### 2.3. Data Sources

National and regional level sales data were kindly provided by IQVIA. The national and regional level population data for the denominators was obtained from the Hungarian Central Statistical Office [25]. National and regional level hospital performance indicators (i.e., patient days) were derived from the National Health Insurance Fund’s (NEAK; Hungarian Acronym) annual statistics [26]. Total patient days in Hungary were used for national level analysis and regional patient days were used for regional data analysis.

The factors that may influence regional variations in opioid use within ambulatory and hospital sectors include population characteristics (percentage of females, percentage of elderly, population, and number of cancer-related deaths), disease prevalence (cancer with International Classification of Diseases Code (ICD) C00-C097 and musculoskeletal conditions with ICD codes M05, M06, M08, M10, M40-43, M45-49, and M80-85), primary hospital care factors (number of general practitioners, case consultations, total hospital bed count, and beds specific to oncology) and socioeconomic variables (GDP, unemployment rates, and nursing allowances). These data were sourced from the Hungarian Central Statistical Office [25] and the National Health Insurance Fund (NEAK) annual reports [26].

### 2.4. Main Outcome Measures

The yearly assessment of opioid usage was conducted using the WHO-defined daily dose system (DDD index, version 2023). Opioid usage was reported as DDD per 1000 inhabitants and per day (DID) for the total utilisation, for ambulatory and hospital care sectors and additionally as DDD per 100 patient days (DHPD), specifically for the hospital sector. The number of admissions and discharges were calculated as one patient day.

### 2.5. Statistical Analysis

Descriptive statistics were shown as mean  ±  standard deviation of the mean (SD), minimum, and maximum for continuous variables. Normality of drug utilisation was assessed by visual interpretations (Q-Q plot). The percent change (% change) reflects the difference between the last year and the first year of the study. We analysed opioid usage trends over a decade. Only data showing usage greater than 0.01 DID and DHPD for various opioids over a minimum of five consecutive years were used in the trend analyses. Linear regression was applied to analyse trends in opioid utilisation throughout the study period. Trends were described by the regression coefficient (average annual change) and significance (*p* value) from the regression formula. A trend was considered significant if *p* < 0.05. Both national and regional-level analyses were conducted. The maximum and minimum ratios were calculated for each region in 2012 and 2021. Correlations between opioid utilisation and regional variables were assessed using a non-parametric test (Spearman’s rank test). Statistical analysis was performed using R 4.2.3. (R Foundation, Vienna, Austria).

## 3. Results

### 3.1. Total Opioid Use in Hungary

Table 1 illustrates the national trends in opioid usage in Hungary for total care, represented in DID and stratified by hospital and ambulatory care sectors. Throughout the 10-year study period, considering total care, there was a general rise in opioid consumption at the national level. Opioid use increased from 4.73 in 2012 to 6.75 DID in 2021. In Hungary, ambulatory care opioid use accounted for more than 90% of total national opioid use during this study period.

### 3.2. Opioid Use in the Ambulatory Care Sector

Nationwide, the ambulatory care sector experienced a rise in opioid use (Table 1 and Table 2). The DID value for opioid use was 4.29 in 2012 and climbed to 6.48 by 2021, marking a 50.90% increase. During this time, oral opioid formulations were primarily utilised (Table 2 and Table S1). The DID value for oral opioid use rose from 3.57 in 2012 to 5.72 in 2021, reflecting a notable growing trend of 60.01%. Meanwhile, the use of transdermal patches experienced a slight, non-significant increase of 7.92% (Table 2). Conversely, the usage of parenteral opioids showed a significant decline of 35.42% (Table 2).

In Hungary, weak opioids were the most commonly used (see Table 2 and Table S2). Weak opioids in the ambulatory care sector exhibited a notable upwards trend (Table 2 and Figure S1) rising from 3.49 DID in 2012 to 5.45 DID in 2021, marking an increase of 61.48%. Although single-component tramadol had the highest usage rate, it also showed a significant annual decline. Conversely, the combination of tramadol-paracetamol escalated from 0.37 DID to 2.17 DID (a 484.61% rise), while tramadol-dexketoprofen increased from 0.12 DID to 0.91 DID (an increase of 650.27%).

Throughout the 10-year study period, the utilisation of strong opioids exhibited a consistent trend (Table 2, Figure S1). Nonetheless, the trends in active agent levels varied: hydromorphone (−87.90%) and morphine (−31.70%) saw a decrease in use, whereas oxycodone demonstrated an upwards trend (176.95%).

### 3.3. Opioid Use in the Hospital Care Sector (DID Unit)

In Hungarian hospitals, a notable decrease in opioid use was recorded, with the DID value falling from 0.44 in 2012 to 0.27 in 2021, representing a substantial decline of 38.48%. When categorised by the route of administration, significant reductions were observed for both parenteral and oral formulations (see Table 3 and Figure S2). The transdermal route also experienced a decreasing trend, though it was not statistically significant. Rectal opioid products remained infrequently used. Moreover, oral opioids dominated in hospital care, increasing their share from 56.4% in 2012 to 60.12% in 2021 (see Table S3).

In the hospital care sector, weak opioid use dominated (see Table S4) but showed a notable decline; their usage dropped from 0.29 DID in 2012 to 0.18 DID in 2021 (−38.02%). Similarly, the usage of single-component tramadol fell significantly (−59.08%) after 2018 (see Table 3), although it remained the most commonly used opioid in 2021. Conversely, the use of weak opioid combinations increased dramatically, including codeine-paracetamol (up 312.61%) and tramadol-paracetamol (up 247.31%), with growth starting gradually in 2017. There was also a decline in strong opioid usage, which decreased from a DID value of 0.15 in 2012 to 0.09 in 2021 (−39.42%).

### 3.4. Opioid Use in the Hospital Care Sector (DHPD Unit)

Opioid usage in hospital care was also quantified as DDD per 100 patient days (DHPD), in line with WHO recommendations. The data are shown in Table 4. Throughout the study period, DHPD measurements indicated that opioid use in hospitals remained nearly constant, with values of 8.35 in 2012 and 8.12 in 2021, contrasting with the trends observed in DID.

When stratified by potency, the use of opioids in hospital care primarily consisted of weak opioids (See Table S6). A slight, non-significant decrease in the usage of strong opioids was observed (−4.25%) (see Table 4 and Figure S3). There was a notable reduction in the use of morphine, hydromorphone, and nalbuphine. In contrast, the use of oxycodone and fentanyl showed a significant upwards trend. Specifically, the trends in fentanyl use exhibited variations between DID and DHPD, with a significant increase recorded for DHPD value.

In terms of administration routes, there was a notable increase in the use of transdermal opioid products 20.66% (Table S5). Conversely, parenteral opioid use showed a significant decrease. Oral opioids remained the most commonly used, exhibiting a consistent trend (see Table 4 and Figure S4).

### 3.5. Regional Use of Opioids

#### 3.5.1. Ambulatory Care Sector

Significant interregional differences were observed in opioid usage within the ambulatory care sector, with maximum to minimun ratios ranging from 1.71 to 1.79 between 2012 and 2021 (Table S7 and Figure S5). The disparities for strong opioids were more pronounced, exceeding 2.5-fold (see Table S7), while weak opioids exhibited maximum to minimum ratios of more than 1.5 in both 2012 and 2021. Both years recorded substantial interregional differences in the use of oral and transdermal opioids (see Table S7).

The trend in regional levels of opioid usage, categorised by potency, reflects a similar pattern, highlighting an increase in the consumption of weak opioids (see Figure S6). Additionally, the use of oral opioids demonstrated a rising trend across all regions (see Figure S7).

#### 3.5.2. Hospital Care Sector

In the hospital sector, interregional variations (maximum to minimun ratios) in opioid use calculated in DHPD were about 1.9 in 2012 and exceeded 3 in 2021 (Table S8 and Figure S8). The parenteral administration route exhibited the most significant regional differences, with over a 4-fold variation in 2012 and a 6-fold variation in 2021, as noted in Table S8. The DHPD values for strong opioids displayed a more than a 3-fold regional variation in both 2012 and 2021. In contrast, weak opioids showed nearly a 2-fold regional difference in 2012 and more than a 4-fold difference in 2021 (See Table S8).

Regional opioid use patterns in hospitals, stratified by potency and administration route, exhibited a plateau or a decline across all regions except for Nograd county, (Figures S9 and S10). Since 2019, Nograd County has experienced a rise in the consumption of weak opioids and oral forms (Figures S9 and S10).

#### 3.5.3. Regional Determinants of Opioid Use in the Ambulatory and Hospital Sectors

We also sought to identify a relationship between regional opioid use and various potential factors (Table S9). Our analysis revealed a moderate positive correlation between the percentage of the geriatric population (aged > 65) (r = 0.475; *p* = 0.035) and the unemployment rate (r = 0.546; *p* = 0.014) with opioid use in the ambulatory care sector. Conversely, a moderate negative correlation was observed between the number of general practitioners (r = −0.458; *p* = 0.043) and regional opioid consumption in Hungary’s ambulatory sector (see Table S9). Additionally, a strong correlation existed between GDP (r = −0.669; *p* = 0.001) and opioid use in the ambulatory sector (see Table S9). However, no significant correlation was detected between regional opioid utilisation in hospital care and regional determinants.

## 4. Discussion

In this article, we presented national data on opioid use in Hungary for both healthcare sectors and at regional level using sales data. We found a general increase in opioid use in total care and ambulatory care in Hungary.

At a national level, increasing trends in total care opioid use were also observed in Australia [15], Denmark [14], Taiwan [27], and Israel [28]. Conversely, declining national total care opioid use has been observed in Denmark and Sweden [21].

The use of opioids in the Hungarian ambulatory care sector increased during the study period. A similar rising trend of ambulatory opioid use has been observed in Ireland [13] and Australia [29]. The scale of opioid use in the ambulatory sector in Hungary, compared to other countries, such as Ireland [13] and Australia, was much lower [29]. In 2019, Ireland’s DID value was 16.68 [13]; in contrast, our research found a DID of 6.04 in 2019, representing a 93.66% lower value. The DID value in Australia in 2015 was 14.0 [15], whereas in Hungary in 2015, it was 4.80, a 97.87% lower value. This confirms that opioid use in Hungarian ambulatory care is lower compared to other countries.

When contrasting this study with earlier Hungarian research, the DID value for opioid use was 6.5 DID in 2020, compared to 5.31 DID found in prior research (20% higher). This variance resulted from using distinct data sources: this study utilised sales data and included in non-reimbursed drugs, including non-reimbursed tramadol products, in the assessment.

Oral opioid use in the Hungarian ambulatory care sector, showed a gradual and notable increase over the years studied. Meanwhile, transdermal opioid use experienced a slight rise. In Ireland, oral opioids were also predominant; however, their usage remained steady, while transdermal opioids saw a growing trend in the ambulatory care sector [13]. The use of oral and transdermal opioids in Australia also increased and oral preparation was the most used [29].

In ambulatory care, the usage of strong opioids has remained unchanged, while there has been a significant rise in the use of weak opioids such as dihydrocodeine, codeine-paracetamol combinations, tramadol-paracetamol combinations, and tramadol-dexketoprofen combinations. Our DID value for weak opioids surpassed the previous finding by Engi, who reported a DID of 4.45 for weak opioids in 2020 [4]; in our study, it was 5.63 (23% higher). In Canada, a decreasing trend in strong and weak opioid (fentanyl, hydromorphone, hydrocodone, morphine, oxycodone, codeine) use in the ambulatory sector in 2020 was reported; however, the trend was already decreasing before the COVID-19 pandemic [30]. Other countries reported different results, for example, an increase in the use of strong opioids, as seen in Ireland [13] and the UK [31]. Nonetheless, Norris classified tramadol as a strong opioid [13], as stated in the British National Formulary [32], and this hampers comparison.

From 2012 to 2021, single-component tramadol dominated the top list of opioid use in Hungary, although its use decreased every year. Tramadol had the highest utilisation among opioids in Ireland [13] and France [33], yet the use of a single component of tramadol has been gradually declining in both countries. In countries like Australia, codeine had a higher utilisation than tramadol [15]. In Hungary, the codeine monotherapy product was solely indicated as an antitussive agent, restricting its use. The dominance of weak opioid utilisation in the Hungarian ambulatory care sector (95.98% in 2021) might be connected to the strict national opioid prescription policy for strong opioids. In Hungary, all opioid medications require a prescription, and strong opioids are designated as narcotic substances prescriptions. In ambulatory care, they can be prescribed for a specified maximum duration.

During the study years, opioid use in the Hungarian hospital care sector declined when measured in DID while remained stable when assessed with DHPD. We faced difficulties in comparing our hospital care data with findings from other studies due to the varying measurement units and study settings. For instance, a study analysing national opioid use in China’s hospital sector indicated a rising trend when expressed in DDD [34]. However, unlike our study, this study did not standardise the nominator by population or hospital activity index, making it difficult to compare the findings directly. Other research has assessed opioid utilisation expressed as DHPD in China [35] and Spain [36] in single hospital studies. Both of these single hospital studies in China and Spain showed increasing opioid use.

In Hungary, oral opioids have been the predominant choice in hospital care. Nonetheless, overall opioid use, regardless of administration route, has been on the decline, except for transdermal opioids, which have seen an increasing trend, as indicated by DHPD. In Australia, oral opioids have been the favoured administration route in both hospital and ambulatory sectors since 1990, while the use of transdermal opioids began to rise after 2008 [29]. In Australia, fentanyl patches have been utilised for chronic cancer pain since 1999. In 2006, the indications was broadened to cover non-cancer pain, resulting in a notable rise in the usage of fentanyl patches in both hospital and outpatient settings [29]. In Hungary, similar events occurred due to the expanding indications of opioid use. The growing popularity of the transdermal route can be linked to its user-friendly design and convenience and its potential to reduce gastrointestinal side effects compared to oral opioids [37,38].

Other researchers analysing hospital data have focused solely on the use of individual opioid agents, without potency stratification, which hampers comparison. Like in ambulatory care, the single component tramadol products displayed the highest utilisation value in hospital care, with a decreasing trend every year. In parallel, the use of weak opioid combinations in Hungarian hospitals has risen sharply since 2017. The doctors may opt for a weak opioid combination since studies indicate that combining NSAIDs and opioids for postoperative pain management reduces nausea, vomiting, and sedation compared to using opioids on their own, while offering improved pain relief [39]. In other countries like Spain [40] and Denmark [14], single-component tramadol use has increased in hospital care from 2010 to 2018.

Regional data highlighted disparities in opioid usage across both sectors. The disparities in opioid use within the Hungarian hospital care sector were more pronounced compared to those in ambulatory care. Jones et al. also found an interregional difference in opioid use within Canada’s ambulatory sector [41]. Our data revealed a positive relationship between age and opioid use, indicating that regions with larger proportion of elderly populations tend to have higher opioid use. Persistent pain is highly prevalent in the elderly population [42]. Consequently, analgesic use, including opioids, might be more frequent among geriatric patients [43].

Women have a higher risk of developing chronic pain [44], as female nociception differs from male nociception, resulting in increased pain sensitivity in women [45]. We assumed there might be a relationship between opioid use and gender; however, no significant correlation was found.

Conversely, the negative correlation between the number of general practitioners and opioid usage implies that increased physician availability leads to reduced opioid prescribing.

A study on US patients with pancreatic cancer found an association between opioid use and individuals undergoing palliative care to alleviate intense pain [46]. Nevertheless, we found no significant correlation between cancer prevalence and opioid use. This is not surprising as opioids are only used as third line agents in pain relief and we did not have patient-level data to stratify the cancer stage and corresponding pain intensity.

Additionally, the relationship between opioid use and economic factors—including a negative correlation with GDP and a positive correlation with unemployment rates—can further explain regional differences. A study in Norway identified that low income and education levels were linked to continued opioid usage [47]. No link was identified between hospital opioid use in different regions and regional factors. Additional research might investigate other elements, including the viewpoints of healthcare professionals and patients.

We used aggregated national-level sales data instead of patient-level data. This could lead to an overestimation of actual opioid usage, as not all sold products may be consumed by patients. Additionally, using aggregated data did not allow us to assess the quality of prescribing. The determinants of regional differences could not be further explored due to the lack of publicly available regional level data. Moreover, we did not use oral morphine equivalent as another metric to assess opioid use.

## 5. Conclusions

Opioid use increased in Hungarian ambulatory care and total care (notably for weak and oral opioids), while remained steady in hospital sector. Regional variations were substantial in both sectors and possibly linked to demographic and economic factors in ambulatory care.

## Figures and Tables

**Table 1:** Opioid Utilisation in Hungary in the Hospital and Ambulatory Care Sectors between 2012 and 2021 expressed as DDD per 1000 inhabitants and per day (DID) and as a percentage of total use.

	2012	2013	2014	2015	2016	2017	2018	2019	2020	2021
Hospital	0.44 (9.31%)	0.45 (9.01%)	0.45 (8.91%)	0.43 (8.17%)	0.43 (8.13%)	0.45 (7.8%)	0.44 (7.11%)	0.41 (6.3%)	0.33 (4.87%)	0.27 (4.02%)
Ambulatory	4.29 (90.69%)	4.54 (90.99%)	4.63 (91.09%)	4.8 (91.83%)	4.88 (91.87%)	5.31 (92.2%)	5.77 (92.89%)	6.04 (93.7%)	6.5 (95.13%)	6.48 (95.98%)
Total	4.73 (100%)	4.99 (100%)	5.08 (100%)	5.22 (100%)	5.31 (100%)	5.76 (100%)	6.21 (100%)	6.45 (100%)	6.83 (100%)	6.75 (100%)

**Table 2:** Opioid use in the Hungarian ambulatory care sector (DDD/1000 inhabitants/day, DID).

Opioid N02A	Year										% Change	Line Plot	Average Annual Change ^b^	*p* ^c^
Administration Route	2012	2013	2014	2015	2016	2017	2018	2019	2020	2021				
Oral	3.57	3.78	3.83	3.99	4.05	4.48	4.94	5.22	5.70	5.72	60.01	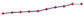	0.261	<0.001 *
Parenteral	0.02	0.02	0.02	0.02	0.02	0.02	0.02	0.02	0.02	0.02	−35.42	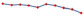	−0.001	0.005 *
Rectal	a	a	a	a	a	a	a	a						
Transdermal	0.69	0.74	0.77	0.78	0.80	0.81	0.80	0.80	0.78	0.75	7.92	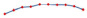	0.006	0.132
Total	4.29	4.54	4.63	4.80	4.88	5.31	5.77	6.04	6.50	6.48	50.90	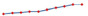	0.266	<0.001 *
Potency														
Strong Opioid														
N02AA01—MORPHINE	0.02	0.02	0.02	0.02	0.02	0.02	0.01	0.01	0.01	0.01	−31.70	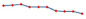	−0.001	<0.001 *
N02AA03—HYDROMORPHONE	0.07	0.06	0.05	0.03	0.03	0.02	0.02	0.02	0.01	0.01	−87.90	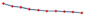	−0.007	<0.001 *
N02AA05—OXYCODONE	0.02	0.03	0.04	0.06	0.06	0.06	0.07	0.06	0.06	0.06	176.95	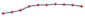	0.004	0.007 *
N02AA55—OXYCODONE COMBINATIONS									a	a				
N02AB02—PETHIDINE	a	a	a	a	a	a	a	a	a	a				
N02AB03—FENTANYL	0.69	0.73	0.77	0.78	0.80	0.81	0.80	0.80	0.78	0.74	7.75	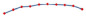	0.006	0.143
N02AE01—BUPRENORPHINE	a	a	a	a	a	a	a	a	a	a				
N02AF02—NALBUFINE	a	a	a	a	a	a	a	a	a	a				
N02AX06—TAPENTADOL					a	a	a							
Total Strong Opioid	0.80	0.84	0.87	0.88	0.91	0.91	0.90	0.89	0.87	0.83	3.08	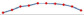	0.004	0.379
Weak Opioid														
N02AA08—DIHYDROCODEINE	0.03	0.03	0.03	0.04	0.04	0.04	0.04	0.05	0.05	0.04	18.47	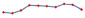	0.001	0.053
N02AJ06—CODEINE AND PARACETAMOL	0.02	0.02	0.02	0.02	0.02	0.02	0.02	0.02	0.02	0.02	−12.04	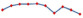	0.000	0.105
N02AJ13—TRAMADOL & PARACETAMOL	0.37	0.37	0.37	0.38	0.39	0.98	1.38	1.72	1.95	2.17	484.61	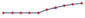	0.227	<0.001 *
N02AJ14—TRAMADOL & DEXKETOPROFEN						0.12	0.44	0.66	0.78	0.91	650.27	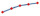	0.192	0.004 *
N02AX02—TRAMADOL	3.07	3.28	3.33	3.46	3.52	3.24	2.99	2.71	2.84	2.52	−17.96	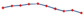	−0.078	0.02 *
Total Weak Opioid	3.49	3.70	3.75	3.91	3.97	4.40	4.86	5.15	5.63	5.65	61.88	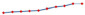	0.262	<0.001 *

a < 0.01 DDD/1000 inhabitants/day, DID. ^b^ Percental change between the last year of the study period and the first study year. ^c^ *p* value refers to the significance of the regression coefficient. * *p* < 0.05 was considered as significant.

**Table 3:** Opioid use in the Hungarian hospital care sector (DDD/1000 inhabitants/day, DID).

Opioid N02A	Year										% Change	Line Plot	Average Annual Change ^b^	*p* ^c^
Administration Route	2012	2013	2014	2015	2016	2017	2018	2019	2020	2021				
Oral	0.25	0.25	0.24	0.23	0.23	0.24	0.26	0.24	0.20	0.16	−34.42	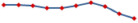	−0.006	0.050
Parenteral	0.10	0.10	0.11	0.10	0.11	0.10	0.08	0.06	0.06	0.04	−62.19	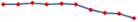	−0.007	0.001 *
Rectal	a	a	a	a	a	a	a							
Transdermal	0.09	0.10	0.10	0.09	0.10	0.10	0.10	0.10	0.08	0.07	−23.65	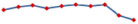	−0.002	0.137
Total	0.44	0.45	0.45	0.43	0.43	0.45	0.44	0.41	0.33	0.27	−38.48	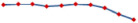	−0.015	0.01 *
Potency														
Strong Opioid														
N02AA01—MORPHINE	0.03	0.03	0.04	0.03	0.03	0.03	0.03	0.02	0.02	0.01	−61.41	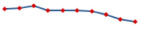	−0.002	0.001 *
N02AA03—HYDROMORPHONE	a	a	a	a	a	a	a	a	a	a				
N02AA05—OXYCODONE	a	a	a	a	a	a	a	a	a	a				
N02AA55—OXYCODONE COMBINATIONS									a	a				
N02AB02—PETHIDINE	a	a	a	a	a	a	a	a	a	a				
N02AB03—FENTANYL	0.09	0.10	0.10	0.09	0.10	0.10	0.10	0.10	0.08	0.07	−23.14	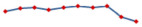	−0.002	0.147
N02AE01—BUPRENORPHINE	a	a	a	a	a	a	a	a	a	a				
N02AF02—NALBUFINE	a	a	a	a	a	a	a	a	a	a				
N02AG01—MORPHINE AND ANTISPASMODICS									a	a				
N02AX06—TAPENTADOL					a									
Total Strong Opioid	0.15	0.15	0.16	0.15	0.15	0.15	0.14	0.14	0.11	0.09	−39.42	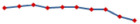	−0.006	0.006 *
Weak Opioid														
N02AA08—DIHYDROCODEINE	a	a	a	a	a	a	a	a	a	a				
N02AJ06—CODEINE AND PARACETAMOL	a	a	a	a	a	a	a	a	a	a				
N02AJ13—TRAMADOL & PARACETAMOL	0.01	a	0.01	0.01	0.01	0.02	0.04	0.03	0.03	0.03	247.31	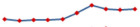	0.004	0.002 *
N02AJ14—TRAMADOL & DEXKETOPROFEN						0.01	0.04	0.04	0.03	0.03	348.20	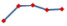	0.004	0.388
N02AX02—TRAMADOL	0.28	0.29	0.28	0.27	0.27	0.26	0.22	0.19	0.16	0.11	−59.08	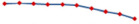	−0.018	<0.001 *
Total Weak Opioid	0.29	0.30	0.29	0.28	0.28	0.30	0.30	0.27	0.23	0.18	−38.02	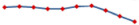	−0.009	0.018 *

a < 0.01 DDD/1000 inhabitants/day, DID. ^b^ Percental change between the last year of the study period and the first study year. ^c^ *p* value refers to the significance of the regression coefficient. * *p* < 0.05 was considered as significant.

**Table 4:** Opioid use in the Hungarian hospital care sector (DDD/100 patients per day, DHPD).

Opioid N02A	Year										% Change	Line Plot	Average Annual Change ^b^	*p* ^c^
Administration Route	2012	2013	2014	2015	2016	2017	2018	2019	2020	2021				
Oral	4.71	4.70	4.58	4.43	4.35	4.79	5.24	4.83	5.20	4.88	3.64	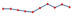	0.055	0.079
Parenteral	1.89	1.94	2.04	1.98	2.03	2.01	1.53	1.26	1.54	1.13	−40.25	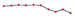	−0.090	0.006 *
Rectal	a	a	a	a	a	a	a							
Transdermal	1.75	1.87	1.91	1.81	1.91	2.03	1.98	2.07	2.13	2.11	20.66	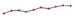	0.039	<0.001 *
Total	8.35	8.51	8.53	8.22	8.30	8.83	8.74	8.16	8.86	8.12	−2.78	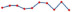	0.004	0.914
Potency														
Strong Opioid														
N02AA01—MORPHINE	0.59	0.62	0.68	0.56	0.56	0.56	0.54	0.46	0.43	0.36	−39.01	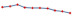	−0.028	0.001 *
N02AA03—HYDROMORPHONE	0.14	0.12	0.13	0.08	0.05	0.03	0.03	0.03	0.03	0.01	−89.30	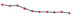	−0.015	<0.001 *
N02AA05—OXYCODONE	0.02	0.03	0.06	0.09	0.09	0.09	0.09	0.08	0.09	0.07	309.66	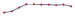	0.006	0.029 *
N02AA55—OXYCODONE COMBINATIONS									a	a				
N02AB02—PETHIDINE	0.08	0.08	0.08	0.07	0.07	0.08	0.06	0.07	0.08	0.05	−40.12	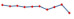	−0.002	0.112
N02AB03—FENTANYL	1.73	1.87	1.90	1.81	1.91	2.03	1.98	2.06	2.12	2.10	21.47	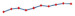	0.040	<0.001 *
N02AE01—BUPRENORPHINE	a	a	a	a	a	a	a	a	a	a				
N02AF02—NALBUFINE	0.21	0.21	0.21	0.20	0.22	0.20	0.05	0.07	0.08	0.04	−79.95	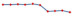	−0.022	0.002 *
N02AG01—MORPHINE AND ANTISPASMODICS									a	a				
N02AX06—TAPENTADOL					a									
Total Strong Opioid	2.78	2.92	3.06	2.83	2.90	2.98	2.75	2.78	2.83	2.67	−4.25	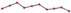	−0.020	0.135
Weak Opioid														
N02AA08—DIHYDROCODEINE	0.09	0.09	0.10	0.11	0.10	0.09	0.10	0.08	0.13	0.15	65.14	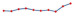	0.004	0.061
N02AJ06—CODEINE AND PARACETAMOL	a	a	a	a	a	a	a	a	a	a				
N02AJ13—TRAMADOL & PARACETAMOL	0.16	0.09	0.10	0.11	0.14	0.42	0.75	0.59	0.76	0.88	448.88	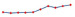	0.096	<0.001 *
N02AJ14—TRAMADOL & DEXKETOPROFEN						0.14	0.77	0.83	0.83	0.98	583.33	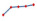	0.173	0.076
N02AX02—TRAMADOL	5.31	5.42	5.26	5.17	5.16	5.18	4.38	3.87	4.31	3.44	−35.32	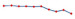	−0.206	<0.001 *
Total Weak Opioid	5.57	5.60	5.47	5.40	5.40	5.84	6.00	5.38	6.03	5.45	−2.04	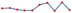	0.023	0.436

a < 0.01 DDD/100 patient per days, DHPD. ^b^ Percental change between the last year of the study period and the first study year. ^c^ *p* value refers to the significance of the regression coefficient. * *p* < 0.05 was considered as significant.

## Data Availability

Data are available from the corresponding author upon reasonable request.

## Supplementary Materials

The following supporting information can be downloaded at: www.mdpi.com/article/10.3390/jcm14030897/s1, Figure S1: Utilisation of opioid potency in Hospital and Ambulatory Sector (DDD/1000 inhabitants/day, DID; Figure S2: Utilisation of opioid administration in Hospital and Ambulatory Sector (DDD/1000 inhabitants/day, DID); Figure S3: Utilisation of opioid potency in Hospital Sector (DDD/100 patients per day, DHPD); Figure S4: Utilisation of opioid administration in Hospital Sector (DDD/100 patients per day, DHPD); Figure S5: Regional difference of ambulatory sector opioid use in Hungary (DID); Figure S6: Regional utilisation of opioid potency in the ambulatory sector (DDD/1000 inhabitants/day, DID); Figure S7: Regional utilisation of opioid potency in the ambulatory sector (DDD/1000 inhabitants/day, DID); Figure S8: Regional difference of hospital sector opioid use in Hungary (DHPD); Figure S9: Regional utilisation of opioid administration in the hospital sector (DDD/100 patients per day, DHPD); Figure S10: Regional utilisation of opioid administration in the hospital sector (DDD/100 patients per day, DHPD); Table S1: Percentual share of the Administration Route in the Ambulatory Care Sector using DDD/1000 inhabitants/day (DID) metrics.; Table S2: Percental share of Opioid Potency in the Ambulatory Care Sector using DDD/1000 inhabitants/day (DID; Table S3: Percentual share of the Administration Route in the Hospital Care Sector using DDD/1000 inhabitants/day (DID) metrics. Table S4: Percental share of Opioid Potency in the Hospital Care Sector using DDD/1000 inhabitants/day (DID); Table S5: Percentual share of the Administration Route in the Hospital Care Sector using DDD/100 patients per day (DHPD) metrics.; Table S6: Percental share of Opioid Potency in the Hospital Care Sector using DDD/100 patients per day (DHPD) metrics; Table S7: Regional difference of opioid use in the Hungarian ambulatory care sector (DID); Table S8: Regional differences of hospital sector opioid use in Hungary (DHPD); Table S9: Association between regional opioid utilisation and regional factors.

## Abbreviations

The following abbreviations are used in this manuscript:

DID	Defined Daily Doses per 1000 Inhabitants per Day
DHPD	DDD per patient per day
MME	Morphine Milligram Equivalent
INCB	International Narcotics Control Board
DDD	Defined Daily Doses
S-DDD	Defined Daily Doses for statistical purposes
WHO	World Health Organisation
ATC	Anatomical Therapeutic Chemical
NEAK	National Health Insurance Fund
ICD	International Classification of Diseases Code
GDP	Gross domestic product
SD	Standard Deviation
NSAIDs	Non-Steroid Anti Inflammation Drugs
GPs	General Practitioners
