# Supplementary material for: Opioid Utilisation in Hungary: National and Regional Analysis in Ambulatory and Hospital Care Sector
Source: J Clin Med. 2025 Jan 29;14(3):897. doi: 10.3390/jcm14030897 (PMC11818432; doi:10.3390/jcm14030897)
Supplement: Supplementary file 1 [file jcm-14-00897-s001.zip › jcm-3411763-supplementary.pdf]

SUPPLEMENTARY MATERIAL

Table S.1 Percentual share of the administration route in the ambulatory care sector using DDD/1000 inhabitants/day (DID) metrics

Sector	Administration	2012	2013	2014	2015	2016	2017	2018	2019	2020	2021
Ambulatory (DID)*	Oral	3.57 (83.22%)	3.78 (83.2%)	3.83 (82.72%)	3.99 (83.23%)	4.05 (83%)	4.48 (84.28%)	4.94 (85.67%)	5.22 (86.45%)	5.7 (87.65%)	5.72 (88.25%)
	Parenteral	0.02 (0.57%)	0.02 (0.51%)	0.02 (0.51%)	0.02 (0.48%)	0.02 (0.42%)	0.02 (0.45%)	0.02 (0.39%)	0.02 (0.34%)	0.02 (0.29%)	0.02 (0.24%)
	Rectal	0.01 (0.12%)	0 (0.1%)	0 (0.1%)	0 (0.09%)	0 (0.08%)	0 (0.07%)	0 (0.02%)	0 (0%)	0 (0%)	0 (0%)
	Transdermal	0.69 (16.09%)	0.74 (16.2%)	0.77 (16.67%)	0.78 (16.21%)	0.8 (16.49%)	0.81 (15.2%)	0.8 (13.91%)	0.8 (13.21%)	0.78 (12.06%)	0.75 (11.51%)
**Total**		4.29 (100%)	4.54 (100%)	4.63 (100%)	4.80 (100%)	4.88 (100%)	5.31 (100%)	5.77 (100%)	6.04 (100%)	6.50 (100%)	6.48 (100%)

*DID: DDD/1000 inhabitants/day

Table S.2 Percental share of opioid potency in the ambulatory care sector using DDD/1000 inhabitants/day (DID) metrics

Sector	Potency	2012	2013	2014	2015	2016	2017	2018	2019	2020	2021
Ambulatory (DID)*	Strong	0.8 (18.68%)	0.84 (18.42%)	0.87 (18.91%)	0.88 (18.43%)	0.91 (18.69%)	0.91 (17.12%)	0.9 (15.68%)	0.89 (14.8%)	0.87 (13.45%)	0.83 (12.76%)
	Weak	3.49 (81.32%)	3.7 (81.58%)	3.75 (81.09%)	3.91 (81.57%)	3.97 (81.31%)	4.4 (82.88%)	4.86 (84.32%)	5.15 (85.2%)	5.63 (86.55%)	5.65 (87.24%)
	Total	4.29 (100%)	4.54 (100%)	4.63 (100%)	4.8 (100%)	4.88 (100%)	5.31 (100%)	5.77 (100%)	6.04 (100%)	6.5 (100%)	6.48 (100%)

*DID: DDD/1000 inhabitants/day

Table S.3 Percentual share of the administration route in the hospital care sector using DDD/1000 inhabitants/day (DID) metrics.

Sector	Administration	2012	2013	2014	2015	2016	2017	2018	2019	2020	2021
Hospital (DID)*	Oral	0.25 (56.4%)	0.25 (55.21%)	0.24 (53.72%)	0.23 (53.86%)	0.23 (52.44%)	0.24 (54.23%)	0.26 (59.9%)	0.24 (59.19%)	0.2 (58.66%)	0.16 (60.12%)
	Parenteral	0.1 (22.65%)	0.1 (22.77%)	0.11 (23.91%)	0.1 (24.05%)	0.11 (24.51%)	0.1 (22.79%)	0.08 (17.48%)	0.06 (15.48%)	0.06 (17.35%)	0.04 (13.92%)
	Rectal	0 (0.04%)	0 (0.02%)	0 (0.04%)	0 (0.04%)	0 (0.03%)	0 (0.03%)	0 (0.01%)	0 (0%)	0 (0%)	0 (0%)
	Transdermal	0.09 (20.92%)	0.1 (22%)	0.1 (22.34%)	0.09 (22.06%)	0.1 (23.01%)	0.1 (22.95%)	0.1 (22.6%)	0.1 (25.33%)	0.08 (23.99%)	0.07 (25.96%)
**Total**		0.44 (100%)	0.45 (100%)	0.45 (100%)	0.43 (100%)	0.43 (100%)	0.45 (100%)	0.44 (100%)	0.41 (100%)	0.33 (100%)	0.27 (100%)

*DID: DDD/1000 inhabitants/day

Table S.4 Percental share of opioid potency in the hospital care sector using DDD/1000 inhabitants/day (DID) metrics.

Sector	Potency	2012	2013	2014	2015	2016	2017	2018	2019	2020	2021
Hospital (DID)*	Strong	0.15 (33.34%)	0.15 (34.28%)	0.16 (35.88%)	0.15 (34.38%)	0.15 (34.93%)	0.15 (33.77%)	0.14 (31.43%)	0.14 (34.08%)	0.11 (31.95%)	0.09 (32.83%)
	Weak	0.29 (66.66%)	0.3 (65.72%)	0.29 (64.12%)	0.28 (65.62%)	0.28 (65.07%)	0.3 (66.23%)	0.3 (68.57%)	0.27 (65.92%)	0.23 (68.05%)	0.18 (67.17%)
	Total	0.44 (100%)	0.45 (100%)	0.45 (100%)	0.43 (100%)	0.43 (100%)	0.45 (100%)	0.44 (100%)	0.41 (100%)	0.33 (100%)	0.27 (100%)

*DID: DDD/1000 inhabitants/day

Table S.5 Percentual share of the administration route in the hospital care sector using DDD/100 patients per day (DHPD) metrics.

Sector	Administration	2012	2013	2014	2015	2016	2017	2018	2019	2020	2021
Hospital (DHPD)*	Oral	4.71 (56.4%)	4.7 (55.21%)	4.58 (53.72%)	4.43 (53.86%)	4.35 (52.44%)	4.79 (54.23%)	5.24 (59.9%)	4.83 (59.19%)	5.2 (58.66%)	4.88 (60.12%)
	Parenteral	1.89 (22.65%)	1.94 (22.77%)	2.04 (23.91%)	1.98 (24.05%)	2.03 (24.51%)	2.01 (22.79%)	1.53 (17.48%)	1.26 (15.48%)	1.54 (17.35%)	1.13 (13.92%)
	Rectal	0 (0.04%)	0 (0.02%)	0 (0.04%)	0 (0.04%)	0 (0.03%)	0 (0.03%)	0 (0.01%)	0 (0%)	0 (0%)	0 (0%)
	Transdermal	1.75 (20.92%)	1.87 (22%)	1.91 (22.34%)	1.81 (22.06%)	1.91 (23.01%)	2.03 (22.95%)	1.98 (22.6%)	2.07 (25.33%)	2.13 (23.99%)	2.11 (25.96%)
**Total**		8.35 (100%)	8.51 (100%)	8.53 (100%)	8.22 (100%)	8.3 (100%)	8.83 (100%)	8.74 (100%)	8.16 (100%)	8.86 (100%)	8.12 (100%)

*DHPD: DDD/100 patients per day

Table S.6 Percental share of opioid potency in the hospital Care DDD/100 patients per day (DHPD) metrics.

Sector	Potency	2012	2013	2014	2015	2016	2017	2018	2019	2020	2021
Hospital (DHPD)*	Strong	2.78 (33.34%)	2.92 (34.28%)	3.06 (35.88%)	2.83 (34.38%)	2.9 (34.93%)	2.98 (33.77%)	2.75 (31.43%)	2.78 (34.08%)	2.83 (31.95%)	2.67 (32.83%)
	Weak	5.57 (66.66%)	5.6 (65.72%)	5.47 (64.12%)	5.4 (65.62%)	5.4 (65.07%)	5.84 (66.23%)	6 (68.57%)	5.38 (65.92%)	6.03 (68.05%)	5.45 (67.17%)
	Total	8.35 (100%)	8.51 (100%)	8.53 (100%)	8.22 (100%)	8.3 (100%)	8.83 (100%)	8.74 (100%)	8.16 (100%)	8.86 (100%)	8.12 (100%)

*DHPD: DDD/100 patients per day

Figure S.1. Utilisation of opioid potency in hospital and ambulatory care sector (DDD/1000 inhabitants/day, DID)

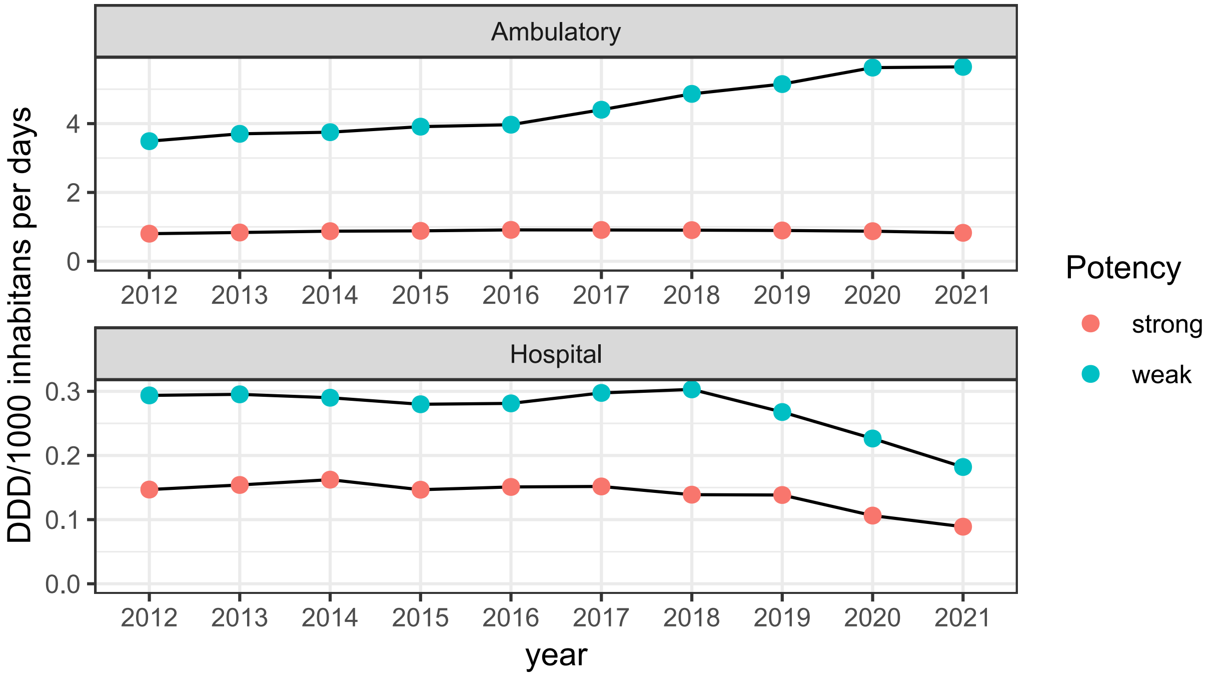

Figure S2. Utilisation of opioid administration in hospital and ambulatory care sector (DDD/1000 inhabitants/day, DID)

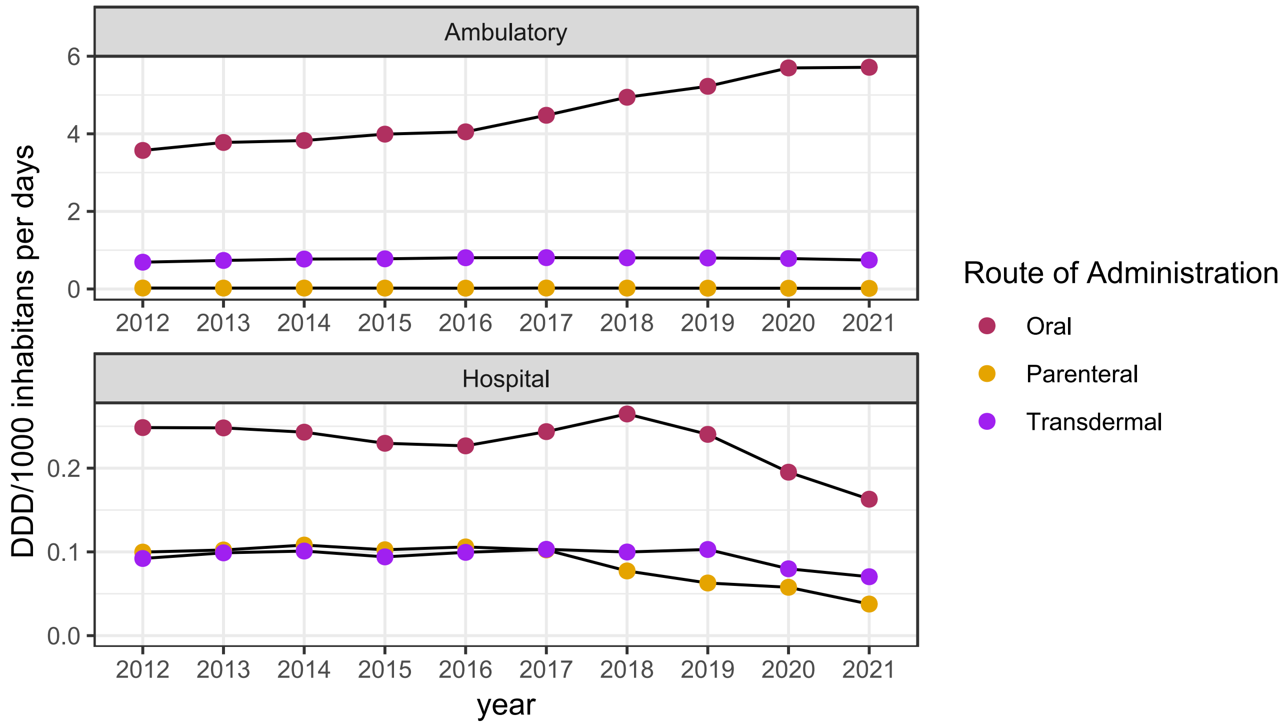

Figure S.3. Utilisation of opioid potency in hospital care sector (DDD/100 patients per day, DHPD)

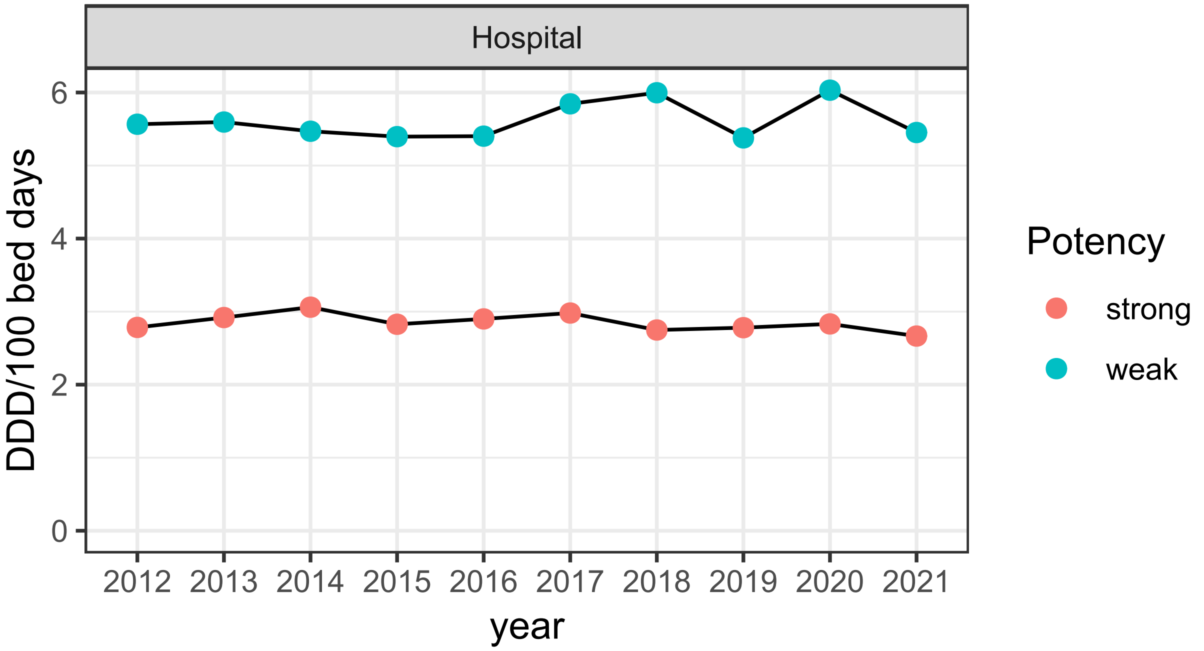

Figure S.4. Utilisation of opioid administration in hospital care sector (DDD/100 patients per day, DHPD)

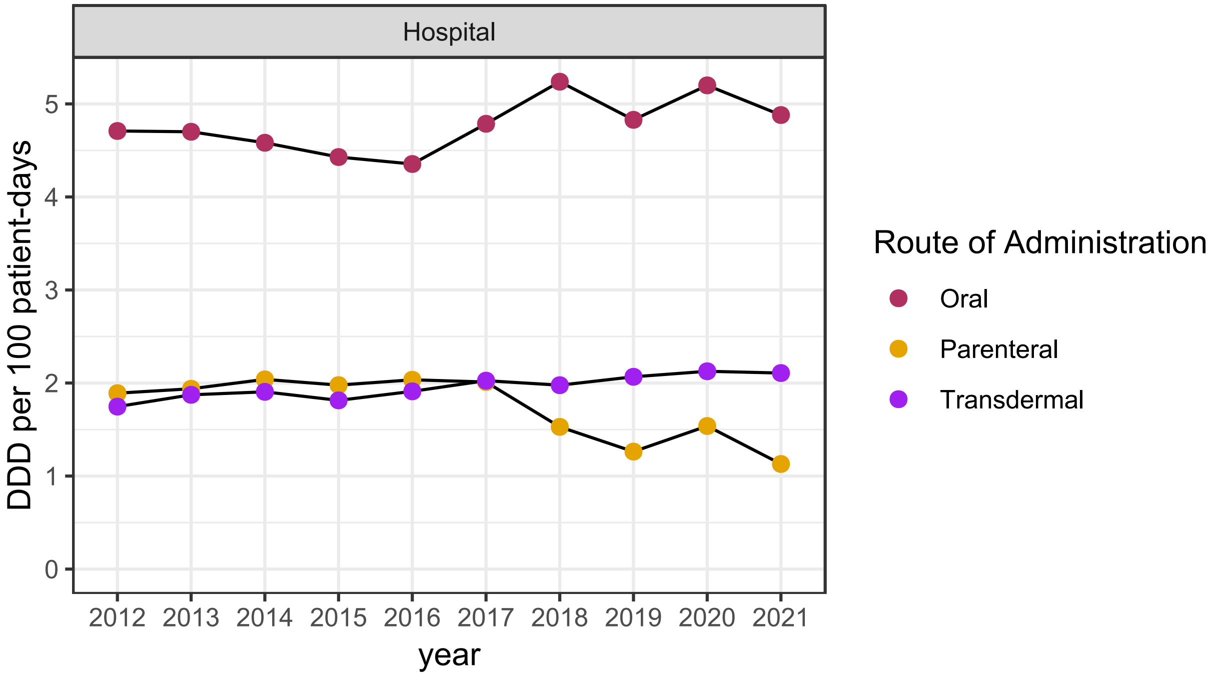

Table S.7. Regional difference of opioid use in the Hungarian ambulatory care sector (DID)

**Opioid N02A**	2012			2021		
**Administration Route**	mean±SD	min-max	ratio max/min	mean±SD	min-max	ratio max/min
Oral	3.74±0.60	2.87-4.76	1.66	6.14±1,17	4.12-7.69	1.87
Parenteral	0.03±0.01	0.01-0.05	5.17	0.01±0.05	0.00-0.02	
Rectal						
Transdermal	0.77±0.23	0.44-1.19	2.72	0.85±0.29	1.48-3.04	3.04
**Total**	4.54±0.70	3.38-5.77	1.71	7.00±1.26	4.78-8.54	1.79
**Potency**						
**Strong Opioid**						
N02AA01 - MORPHINE	0.01±0.01	0.00-0.04		0.01±0.01	0.00-0.04	
N02AA03 - HYDROMORPHONE	0.08±0.03	0.04-0.13	3.22	0.01±0.01	0.00-0.04	
N02AA05 - OXYCODONE	0.02±0.02	0.00-0.08		0.06±0.05	0.01-0.17	12.06
N02AA55 - OXYCODONE COMBINATIONS						
N02AB02 - PETHIDINE						
N02AB03 - FENTANYL	0.77±0.23	0.43-1.19	2.72	0.85±0.29	0.49-1.48	3.04
N02AE01 - BUPRENORPHINE						
N02AF02 - NALBUFINE						
N02AX06 - TAPENTADOL						
**Total**	0.89±0.25	0.51-1.36	2.66	0.93±0.31	0.53-1.55	2.93
**Weak Opioid**						
N02AA08 - DIHYDROCODEINE	0.03±0.01	0.01-0.07	6.43	0.04±0.02	0.01-0.07	5.48
N02AJ06 - CODEINE AND PARACETAMOL	0.02±0.02	0.00-0.07		0.02±0.01	0.00-0.05	
N02AJ13 - TRAMADOL AND PARACETAMOL	0.37±0.15	0.20-0.71	3.52	2.32±0.57	1.34-3.32	2.47
N02AJ14 - TRAMADOL & DEXKETOPROFEN				0.99±0.33	0.62-2.08	3.32
N02AX02 - TRAMADOL	3.24±0.51	2.42-4.29	1.77	2.70±0.51	1.75-3.51	2.01
**Total**	3.66±0.59	2.79-4.65	1.67	6.07±1.16	4.06-7.61	1.88

Figure S.5. Regional difference of ambulatory sector opioid use in Hungary (DID)

2012	2021

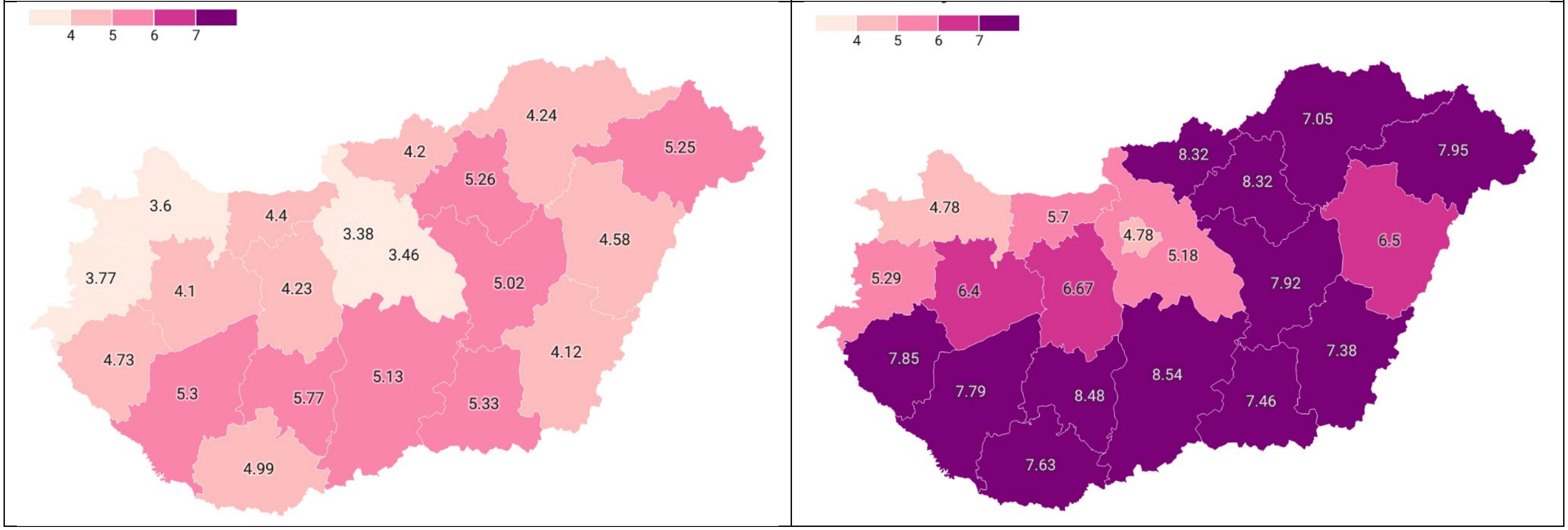

Figure S.6 Regional utilisation of opioid potency in the ambulatory sector (DDD/1000 inhabitants/day, DID)

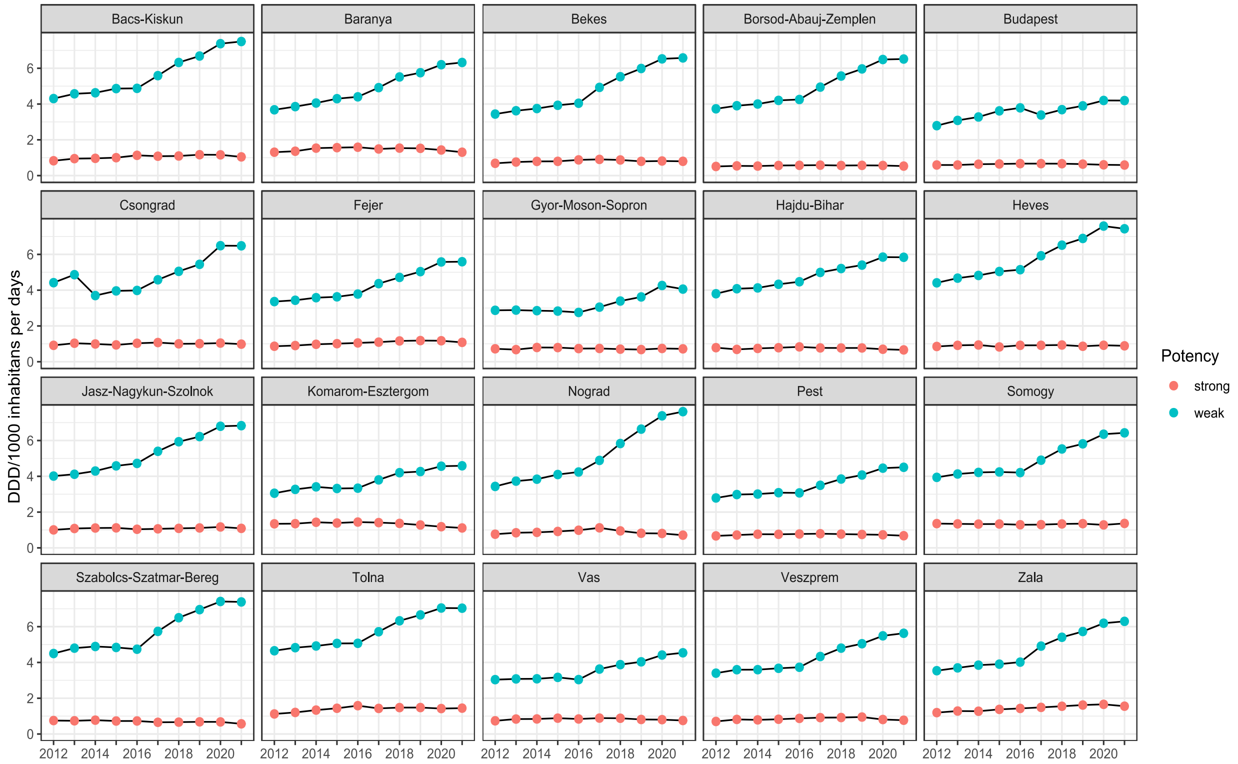

Figure S.7. Regional utilisation of opioid administration in the ambulatory sector (DDD/1000 inhabitants/day, DID)

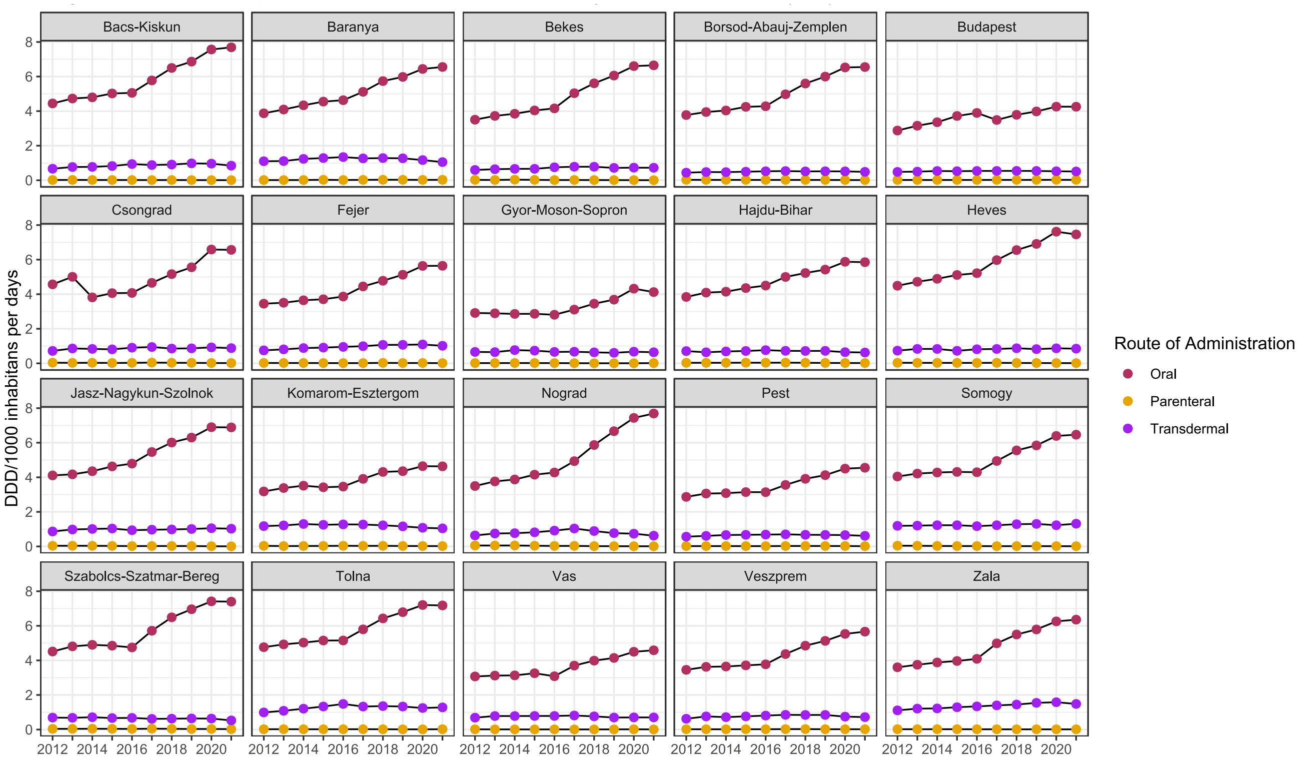

Table S.8. Regional differences of hospital sector opioid use in Hungary (DHPD)

**Opioid N02A**	2012			2021		
**Administration Route**	mean±SD	min-max	ratio max/min	mean±SD	min-max	ratio max/min
Oral	4.63±0.93	2.94-6.11	2.07	5.08±2.53	3.14-15.02	4.78
Parenteral	1.80±0.75	0.76-3.52	4.60	1.03±0.53	0.34-2.36	6.85
Rectal						
Transdermal	1.80±0.55	0.82-2.73	3.31	2.23±0.74	1.31-3.93	3.01
**Total**	8.23±1.40	5.95-11.25	1.89	8.34±2.71	5.71-17.27	3.03
**Potency**						
**Strong Opioid**						
N02AA01 - MORPHINE	0.54±0.40	0.10-1.48	14.48	0.35±0.30	0.03-1.11	41.66
N02AA03 - HYDROMORPHONE	0.13±0.09	0.02-0.30	18.76	0.02±0.02	0.00-0.08	
N02AA05 - OXYCODONE	0.04±0.05	0.00-0.16		0.07±0.06	0.00-0.23	
N02AA55 - OXYCODONE COMBINATIONS				0.03±0.02	0.00-0.07	
N02AB02 - PETHIDINE	0.06±0.04	0.00-0.16		0.04±0.03	0.00-0.10	
N02AB03 - FENTANYL	1.79±0.55	0.82-2.73	3.34	2.22±0.74	1.30-3.93	3.02
N02AE01 - BUPRENORPHINE	0.02±0.04	0.00-0.14		0.01±0.01	0.00-0.05	
N02AF02 - NALBUFINE	0.20±0.15	0.04-0.55	15.74	0.04±0.03	0.00-0.12	
N02AG01 - MORPHINE AND ANTISPASMODICS						
N02AX06 - TAPENTADOL						
**Total**	2.75±0.89	1.48-5.15	3.47	2.74±0.93	1.75-5.26	3.01
**Weak Opioid**						
N02AA08 - DIHYDROCODEINE	0.08±0.06	0.00-0.28		0.15±0.14	0.02-0.54	29.92
N02AJ06 - CODEINE AND PARACETAMOL	0.01±0.01	0.00-0.02		0.02±0.02	0.00-0.03	
N02AJ13 - TRAMADOL AND PARACETAMOL	0.15±0.13	0.01-0.42	51.12	0.85±0.50	0.05-2.27	47.34
N02AJ14 - TRAMADOL & DEXKETOPROFEN				1.34±2.17	0.14-9.89	70.13
N02AX02 - TRAMADOL	5.25±1.02	3.46-6.75	1.95	3.27±0.79	2.24-5.80	2.58
**Total**	5.48±1.05	3.56-6.90	1.94	5.60±2.58	3.58-15.28	4.27

Figure S.8. Regional difference of hospital sector opioid use in Hungary (DHPD)

2012

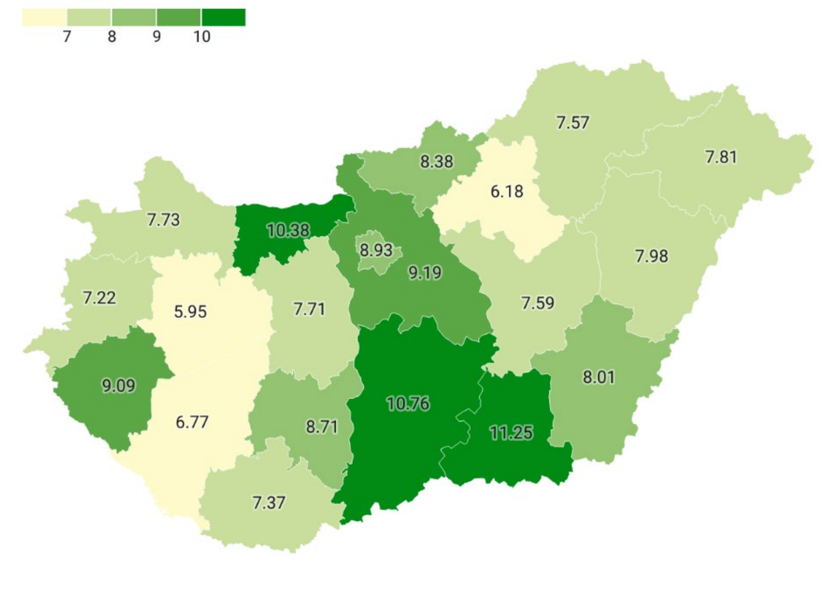

2021

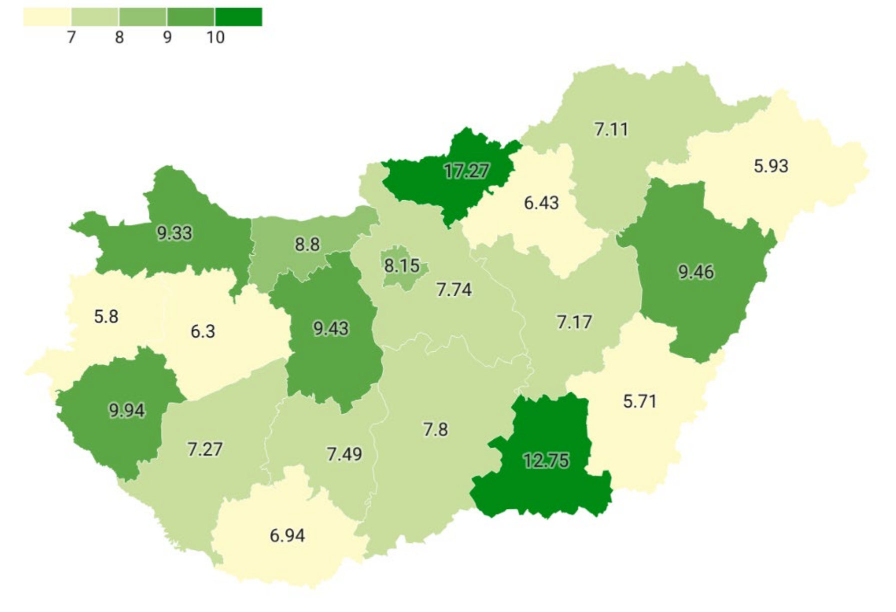

Figure S.9. Regional utilisation of opioid administration in the hospital sector (DDD/100 patients per day, DHPD)

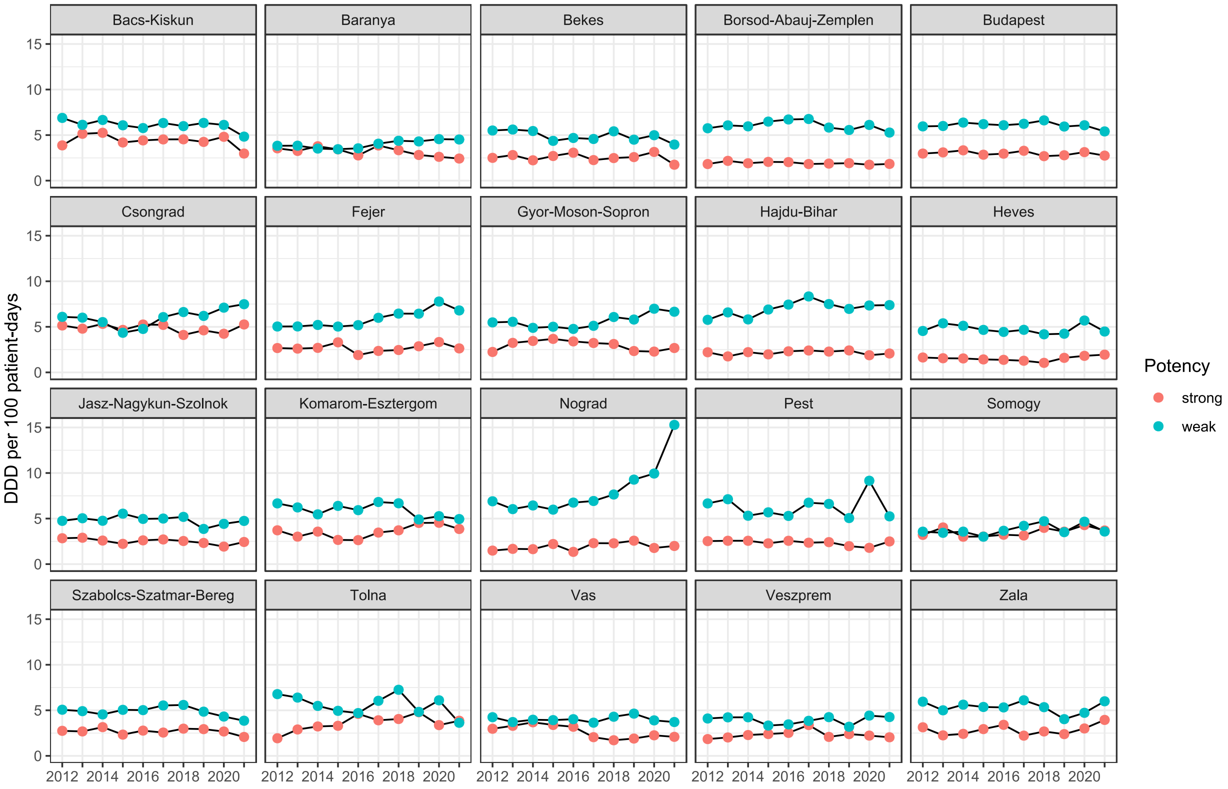

Figure S.10. Regional utilisation of opioid administration in the hospital sector (DDD/100 patients per day, DHPD)

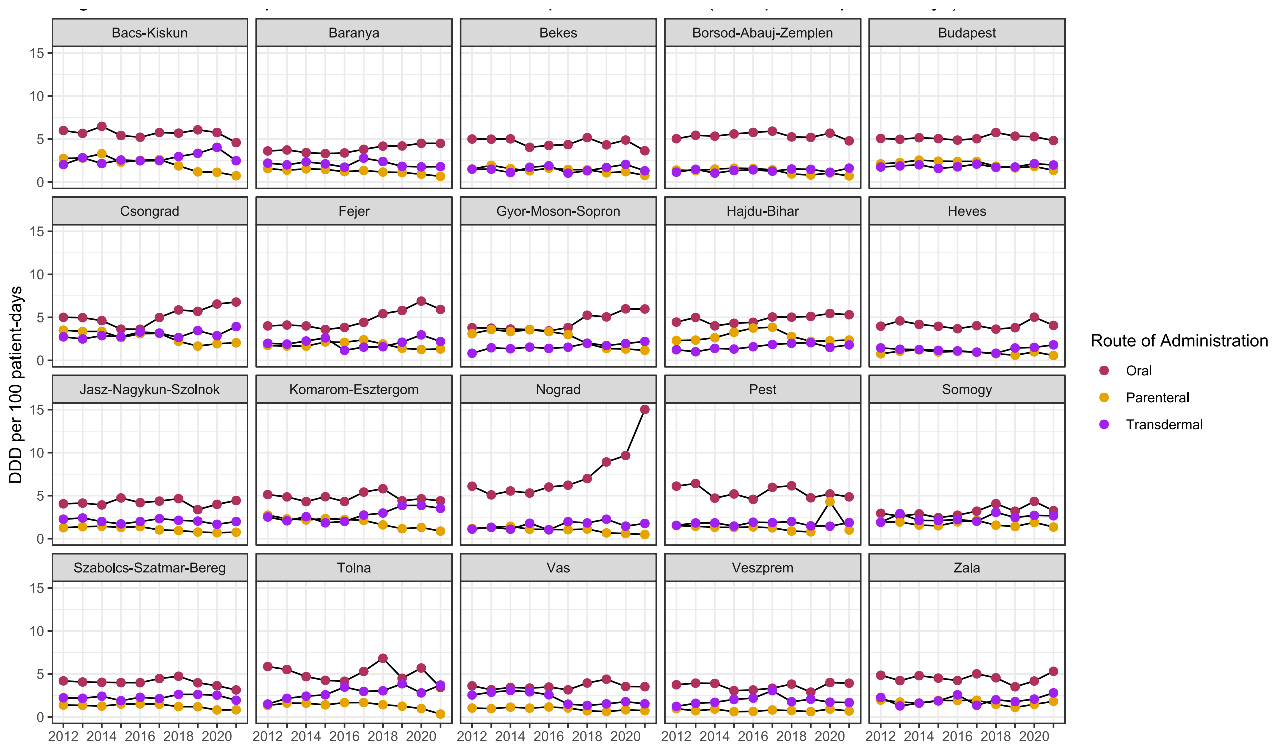

Table S.9. Association between regional opioid utilisation and regional factors

Association between regional opioid utilisation and regional factors		
Factor	Ambulatory DID	Hospital DHPD
	Correlation coefficient (p value)	
Percentage of female population	0.085 (0.719)	0.144 (0.542)
Percentage of geriatric (>65 years old) population	0.475 (0.035) *	0.014 (0.955)
Prevalence of cancer (C00-C97)	0.050 (0.835)	0.194 (0.413)
Prevalence of muskulosketal disease (M05, M06, M08, M10, M40-M43, M45-M49, M80-M85)	0.238 (0.313)	-0.158 (0.506)
Number of general practitioners (capita)	-0.458 (0.043) *	0.093 (0.695)
Number of general practitioners' consultations (cases)	-0.321 (0.166)	-0.031(0.896)
Total hospital bed count (active and chronic)	0.057 (0.811)	-0.006 (0.816)
Number of hospital bed in oncology ward	-0.192 (0.416)	-0.194 (0.412)
GDP	-0.669 (0.001) *	0.245 (0.298)
Average monthly number of recipients of nursing allowance (capita)	0.129 (0.587)	-0.053 (0.826)
Unemployment rate by counties and regions in percent	0.546 (0.014) *	-0.165(0.482)
Death Caused by cancer	-0.329 (0.156)	-0.012 (0.962)

Associations were tested with Spearman’s rank test.

*p values < 0.05 showed statistical significance
